# “Life, Interrupted”: A Patient Experience Encompassing the Journey From Hospital to the Community, With Support From the Mental Health Intensive Support Team (MhIST)

**DOI:** 10.1192/bjo.2022.373

**Published:** 2022-06-20

**Authors:** Jen McPherson, Jonathon Whyler, Amrith Shetty, Lucy Mahon

**Affiliations:** 1 The Open University, Milton Keynes, United Kingdom; 2 Cheshire and Wirral Partnership NHS Foundation Trust, Chester, United Kingdom; 3 Health Education England North West, Manchester, United Kingdom

## Abstract

**Aims:**

We present outcomes of a newly developed Community Rehabilitation team (MhIST) using the context of Jen's personal story. Jen is a 31-year-old student and freelance journalist. This story encompasses her journey from inpatient rehabilitation services to the community, completed with support from MhIST.

**Methods:**

“For nearly four years, I was sectioned under the Mental Health Act as an inpatient in hospital. As I had been denied my fundamental liberties for so long, the prospect of leaving hospital for good and enjoying total freedom was both exhilarating and terrifying. How would I fare in the community, living on my own? Would I be lonely? Would I relapse? Would I survive?”

Upon leaving hospital, I immediately received intensive support from MhIST. They were the bridge between the gulf that was hospital and the community. Since leaving hospital, I have been relishing my freedom. I enjoy meeting up with my friends after so long apart. I have volunteered at The Storyhouse, a local arts venue. The Spider Project – a non-clinical community mental health service in Chester - has also provided me with fulfilling activities from yoga to creative writing. The MHIST team have not only kept me well but, most importantly, helped me thrive. Leaving hospital has been an adventure. It has been a joy to regain my independence and freedom. To live rather than to exist. Life is amazing. Long may it continue.”

**Results:**

MhIST provides an intensive rehabilitation and recovery service, delivering bespoke packages of care to individuals. This is achieved using key working and a shared team approach, outcome focused goal-based interventions, weekly reflective/formulation meetings, and a focus on social rehabilitation. Patients referred to MhIST will have a high level of complexity plus severe, treatment refractory symptoms, with impaired social, interpersonal and occupational function and high support needs. They may have co-occurring mental health conditions including substance misuse or neurodevelopmental disorders.

MhIST is a new service and has been active for around 6 months. The first 10 patients referred have been from acute wards (3), community mental health teams (1), and inpatient rehabilitation wards (6). 60% of patients are currently housed in independent accommodation.

**Conclusion:**

Jen's story narrates the experience she encountered during transition from inpatient rehabilitation services to the community. This was completed with support from MhIST, a new community rehabilitation service which provides an intensive rehabilitation and recovery service.

